# Supplementary material for: Active droplets through enzyme-free, dynamic phosphorylation
Source: Nat Commun. 2024 May 17;15:4204. doi: 10.1038/s41467-024-48571-z (PMC11101487; doi:10.1038/s41467-024-48571-z)
Supplement: Supplementary file 1 — Supplementary Information [file 41467_2024_48571_MOESM1_ESM.pdf]

Supplementary Information for:

Title: Active droplets through enzyme-free, dynamic phosphorylation

Authors: Simone M. Poprawa,[1] Michele Stasi,[1] Brigitte A. K. Kriebisch,[1] Monika Wenisch,[1] Judit Sastre,[1] Job Boekhoven[1]

Affiliations:

[1] Department of Bioscience, Technical University of Munich, Lichtenbergstrasse 4, 85748 Garching, Germany

Table of Contents

I. Supplementary Methods 3

(A) Characterization of the synthesized compounds. 3

(B) Kinetic model 4

II. Supplementary Tables 12

III. Supplementary Figures 17

I. Supplementary Methods

(A) Characterization of the synthesized compounds.

Characterization of MAP.

^{31}P-NMR (D_2O, pH 6.5, 500 MHz): δ -3.88

Elemental analysis:

theoretical values: H: 2.24%; N: 10.37%; K: 28.94%; O: 35.53%; P: 22.93%

measured values: H: 2.29%; N: 9.94%; K: 29.3%; P: 21.81%.

Characterization of DAP.

^{31}P-NMR (D_2O, pH 6.5, 500 MHz): δ 12.33

Elemental analysis:

theoretical values: H: 3.42%; N: 23.74%; Na: 19.48%; O: 27.12%; P: 26.25%

measured values: H: 3.62%; N: 21.06%; Na: 18.7%; P: 23.69%.

Characterization of the peptides.

Ac-GHG-OH (Supplementary Fig. 18)	Mass calculated [g mol^{-1}]	311.12
	Mass observed [g mol^{-1}]	312.12 $[M+H]^+$
	Retention time [min]	1.19
Ac-Y(OMe)DHDD-OH (Supplementary Fig. 19)	Mass calculated [g mol^{-1}]	719.24
	Mass observed [g mol^{-1}]	718.25 $[M-H]^-$
	Retention time [min]	14.0 (Calibration factor = 12.717)
Ac-Y(OMe)DHDD-NH_2 (Supplementary Fig. 20)	Mass calculated [g mol^{-1}]	718.26
	Mass observed [g mol^{-1}]	717.47 $[M-H]^-$
	Retention time [min]	11.8 (Calibration factor = 14.093)

(B) Kinetic model

A kinetic model was written in COPASI that described each reaction involved in the chemical reaction network. The concentrations of each reactant were calculated for every 5 minutes in the cycle. The model was used to obtain fitted curves based on ^{31}P-NMR or HPLC data that described the evolution of the concentration of the phosphorylated species, fuel, and waste. The fitted reaction rate constant values of the hydrolysis in the presence of pyridine and of the phosphorylation of His were combined and used while fitting the k-values for the phosphorylation in the presence of pyridine.

The *Levenberg-Marquardt* method was used to obtain the k-values, and the deterministic (LSODA) method was used to obtain the time courses.

The reaction rate constant of the 3-pHis was determined empirically when no other species or fuel were present anymore. Therefore, the predicted and the experimentally measured evolution fit nicely. The hydrolysis of MAP was also calculated empirically, but the other constants (up to 17) were fitted and thus show higher deviations (error of the fit) as there are many combinations possible, also with correlated parameters. Despite that, the fits align very well with the experimental data.

The kinetic model is good for predicting the evolution of a single species and can potentially help to design a self-assembling system. But the reaction rate constants, which are not determined by the data, should not be compared directly.

The Sankey-diagram for the consumption of fuel was generated with the help of the following website: https://sankeymatic.com

Reaction 0 (k_0):

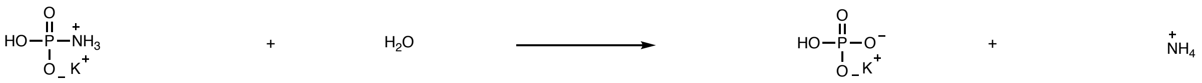

The direct hydrolysis of the fuel (MAP) to inorganic phosphate follows pseudo-first-order kinetics with respect to MAP. The rate constants and the corresponding half-lives are summarized in Supplementary Table 5.

Reaction 1 (k_1):

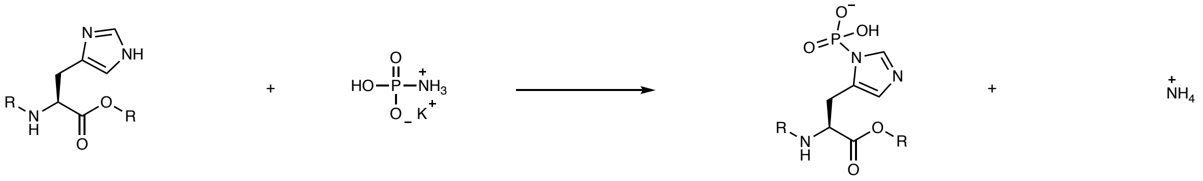

The reaction of MAP with the His-precursor to the 1-pHis-product follows second-order kinetics. The rate constants are shown in Supplementary Table 5.

Reaction -1 (k_{-1}):

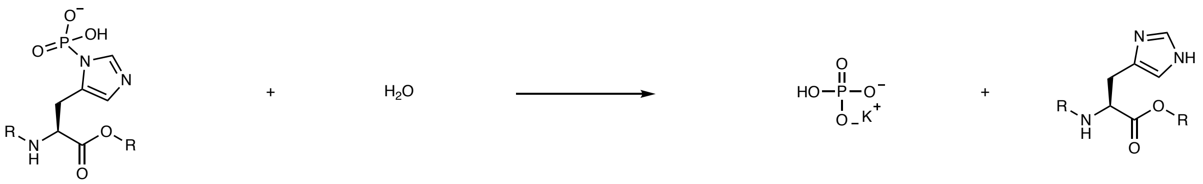

The hydrolysis of 1-pHis follows pseudo-first-order kinetics with respect to 1-pHis. The rate constants are shown in Supplementary Table 5.

Reaction 2 (k_2):

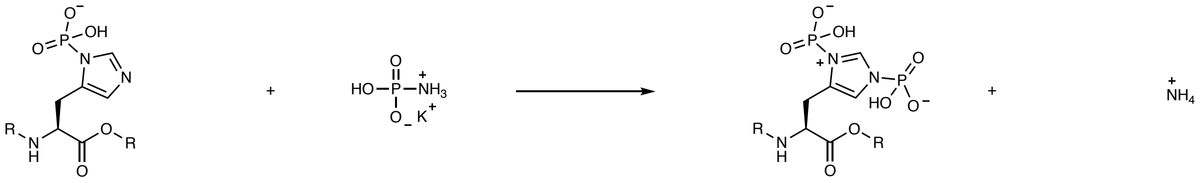

The reaction of MAP with 1-pHis to the 1,3-bpHis-product follows second-order kinetics. The bisphosphorylated species was only observed for histidine and Ac-GHG-OH at pH = 7.5. The rate constants are shown in Supplementary Table 5.

Reaction -2 (k_{-2}):

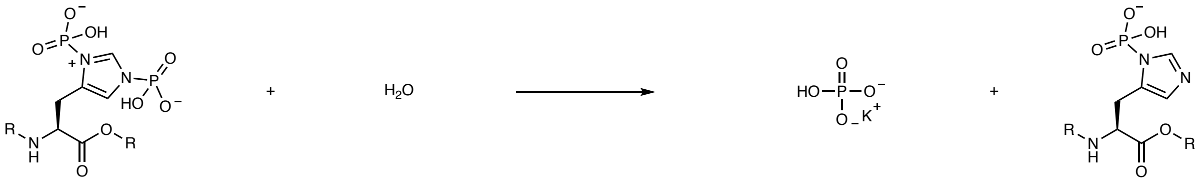

The hydrolysis of 1,3-bispHis to 1-pHis follows pseudo-first-order kinetics with respect to the 1-phospho-group of 1,3-bispHis. The rate constants are shown in Supplementary Table 5.

Reaction 3 (k_3):

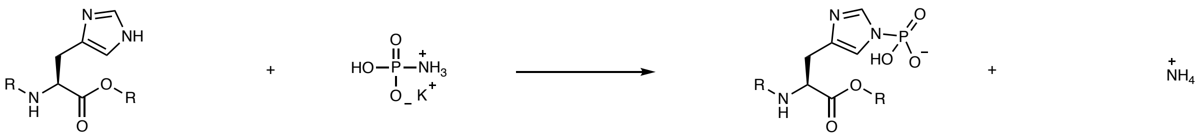

The reaction of MAP with the His-precursor to the 3-pHis-product follows second-order kinetics. The rate constants are shown in Supplementary Table 5.

Reaction -3 (k_{-3}):

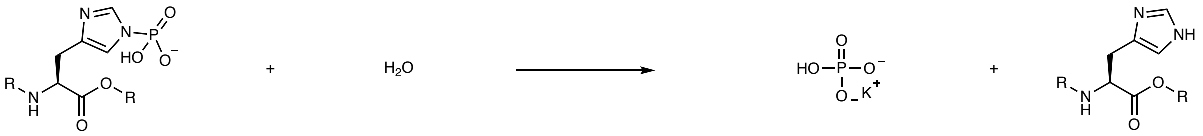

The hydrolysis of 3-pHis follows pseudo-first-order kinetics with respect to 3-pHis. The rate constants for His and Ac-GHG-OH were determined experimentally *via* ^{31}P-NMR by monitoring the concentration profiles after the fuel was consumed. The rate constants are shown in Supplementary Table 5. To compare the evolution of Ac-G(3-pHis)G-OH in the presence of pyridine, the apparent first-order constant $k'_{3\text{-pHis}}$ was introduced. After the fuel was consumed, it was assumed that the hydrolysis follows pseudo-first-order kinetics. The apparent rate constants in the presence of pyridine are summarized in Supplementary Table 7.

Reaction 4 (k_4):

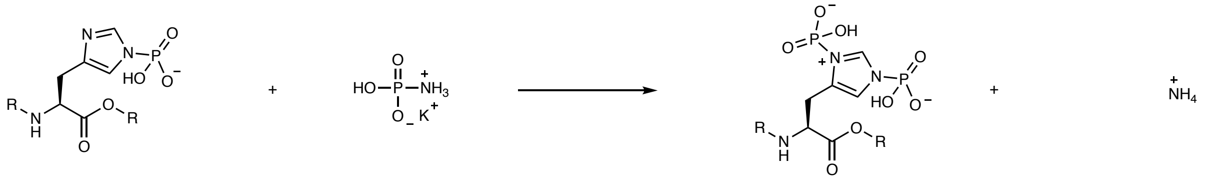

The reaction of MAP with 3-pHis to the 1,3-bpHis-product follows second-order kinetics. The bisphosphorylated species was only observed for His and Ac-GHG-OH at pH = 7.5. The rate constants are shown in Supplementary Table 5.

Reaction -4 (k_{-4}):

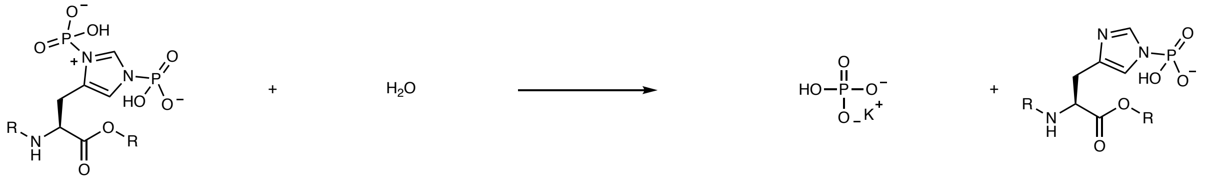

The hydrolysis of 1,3-bpHis to 3-pHis follows pseudo-first-order kinetics with respect to the 3-phospho-group of 1,3-bpHis. The rate constants are shown in Supplementary Table 5.

Reaction py (k_{py}):

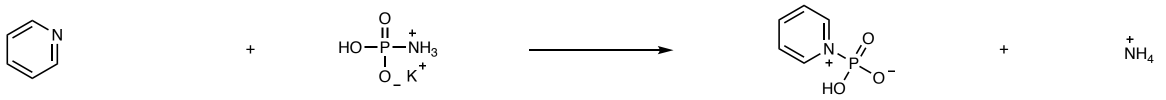

The reaction of MAP with pyridine (Py) to pyridinio-*N*-phosphonate (pPy) follows second-order reaction kinetics. k_{py} values were fitted with COPASI to experimental data. The rate constants are shown in Supplementary Table 5.

Reaction -py (k_{-py}):

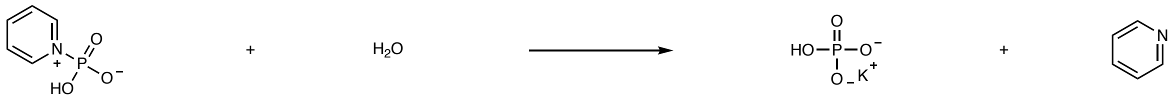

The hydrolysis of pPy to pyridine and phosphate follows pseudo-first-order kinetics with respect to pPy. k_{-py} values were fitted with COPASI to experimental data. The rate constants are shown in Supplementary Table 5.

Reaction 5 (k_5):

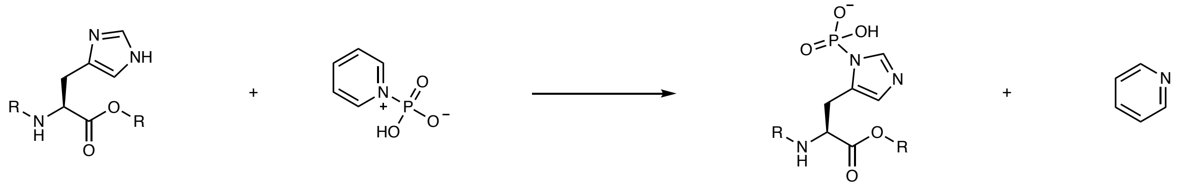

The reaction of pPy with His to the 1-pHis-product follows second-order kinetics. The rate constants are shown in Supplementary Table 5.

Reaction -5 (k_{-5}):

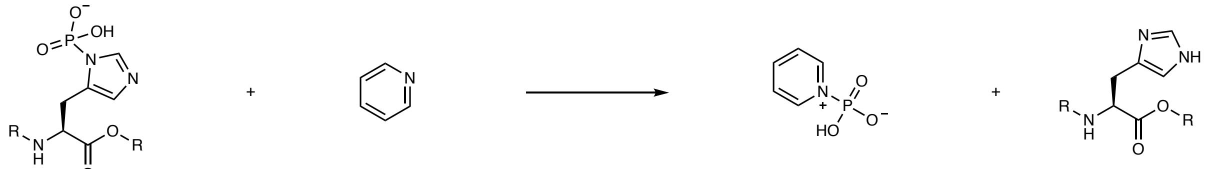

The reaction of 1-pHis with pyridine to form pPy and His follows second-order kinetics. The rate constants are shown in Supplementary Table 5.

Reaction 6 (k_6):

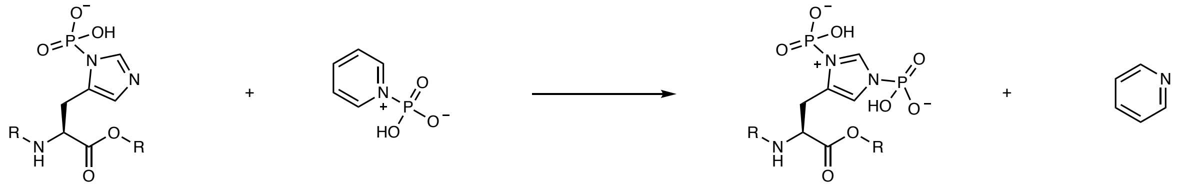

The reaction of pPy with 1-pHis to the 1,3-bpHis-product follows second-order kinetics. The rate constants are shown in Supplementary Table 5.

Reaction -6 (k_{-6}):

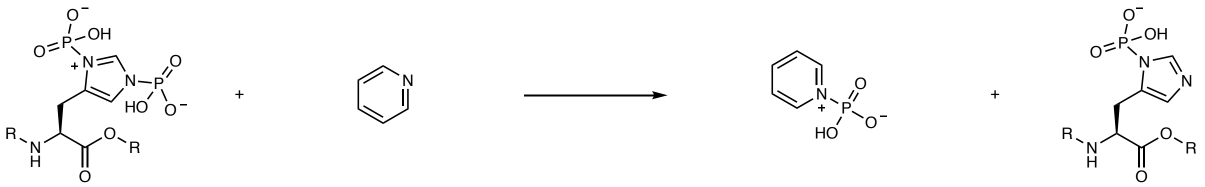

The reaction of 1,3-bpHis with pyridine to form pPy and 1-pHis follows second-order kinetics. The rate constants are shown in Supplementary Table 5.

Reaction 7 (k_7):

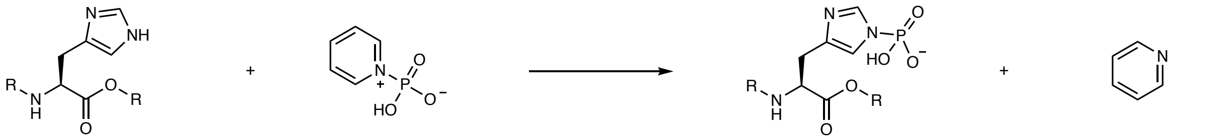

The reaction of pPy with His to the 3-pHis-product follows second-order kinetics. The rate constants are shown in Supplementary Table 5.

Reaction -7 (k_{-7}):

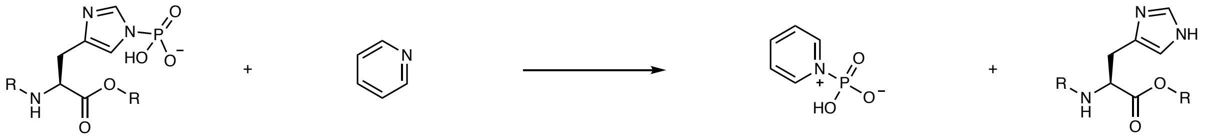

The reaction of 3-pHis with pyridine to form pPy and His follows second-order kinetics. The rate constants are shown in Supplementary Table 5. To compare the evolution of Ac-G(3-pHis)G-OH in the presence of pyridine, the apparent first-order constant $k'_{3\text{-pHis}}$ was introduced. After the fuel was consumed, it was assumed that the hydrolysis follows pseudo-first-order kinetics. The apparent rate constants in the presence of pyridine are summarized in Supplementary Table 7.

Reaction 8 (k_8):

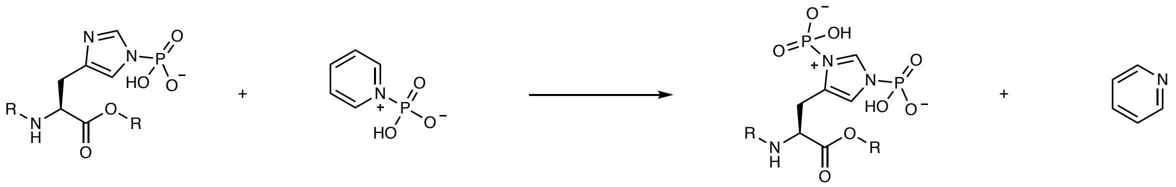

The reaction of pPy with 3-pHis to the 1,3-bpHis-product follows second-order kinetics. The rate constants are shown in Supplementary Table 5.

Reaction -8 (k_{-8}):

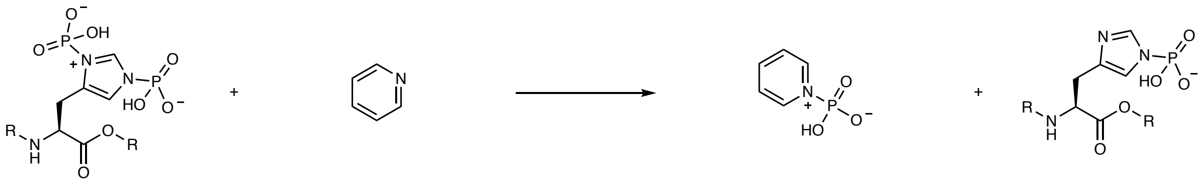

The reaction of 1,3-bpHis with pyridine to form pPy and 3-pHis follows second-order kinetics. The rate constants are shown in Supplementary Table 5.

$$\frac{d[MAP]}{dt} = - k_0[MAP] - k_1[MAP][His] - k_2[MAP][1\text{-}pHis] - k_3[MAP][His] - k_4[MAP][3\text{-}pHis] - k_{Py}[MAP][Py]$$

$$\frac{d[Pi]}{dt} = + k_0[MAP] + k_{-1}[1\text{-}pHis] + k_{-2}[1,3\text{-}bpHis] + k_{-3}[3\text{-}pHis] + k_{-4}[1,3\text{-}bpHis] + k_{-Py}[pPy]$$

$$\frac{d[His]}{dt} = + k_1[MAP][His] + k_{-1}[1\text{-}pHis] - k_3[MAP][His] + k_{-3}[3\text{-}pHis] - k_5[pPy][His] + k_{-5}[Py][1\text{-}pHis] - k_7[pPy][His] + k_{-7}[Py][3\text{-}pHis]$$

$$\frac{d[1\text{-}pHis]}{dt} = + k_1[MAP][His] - k_{-1}[1\text{-}pHis] - k_2[MAP][1\text{-}pHis] + k_{-2}[1,3\text{-}bpHis] + k_5[pPy][His] - k_{-5}[Py][1\text{-}pHis] - k_6[pPy][1\text{-}pHis] + k_{-6}[Py][1,3\text{-}bpHis]$$

$$\frac{d[3\text{-}pHis]}{dt} = + k_3[MAP][His] - k_{-3}[3\text{-}pHis] - k_4[MAP][1\text{-}pHis] + k_{-4}[1,3\text{-}bpHis] + k_7[pPy][His] - k_{-7}[Py][3\text{-}pHis] - k_8[pPy][3\text{-}pHis] + k_{-8}[Py][1,3\text{-}bpHis]$$

$$\frac{d[1,3\text{-}bpHis]}{dt} = + k_2[MAP][1\text{-}pHis] - k_{-2}[1,3\text{-}bpHis] + k_4[MAP][3\text{-}pHis] - k_{-4}[1,3\text{-}bpHis] + k_6[pPy][1\text{-}pHis] - k_{-6}[Py][1,3\text{-}bpHis] + k_8[pPy][3\text{-}pHis] - k_{-8}[Py][1,3\text{-}bpHis]$$

$$\frac{d[Py]}{dt} = - k_{Py}[MAP][Py] + k_{-Py}[pPy] + k_5[pPy][His] - k_{-5}[Py][1\text{-}pHis] + k_7[pPy][His] - k_{-7}[Py][3\text{-}pHis] + k_6[pPy][1\text{-}pHis] - k_{-6}[Py][1,3\text{-}bpHis] + k_8[pPy][3\text{-}pHis] - k_{-8}[Py][1,3\text{-}bpHis]$$

$$\frac{d[pPy]}{dt} = + k_{Py}[MAP][Py] - k_{-Py}[pPy] - k_5[pPy][His] + k_{-5}[Py][1\text{-}pHis] - k_7[pPy][His] + k_{-7}[Py][3\text{-}pHis] - k_6[pPy][1\text{-}pHis] + k_{-6}[Py][1,3\text{-}bpHis] - k_8[pPy][3\text{-}pHis] + k_{-8}[Py][1,3\text{-}bpHis]$$

System of the used differential equations.

II. Supplementary Tables

Supplementary Table 1. Linear gradient for the purification of Ac-GHG-OH tracked at 220 nm.

Time [min]	Flow rate [mL/min]	H_20+TFA 0.1%	ACN+TFA 0.1%
0	20	95%	5%
5	20	80%	20%
7	20	2%	98%
12	20	2%	98%
13	20	95%	5%
15	20	95%	5%

Supplementary Table 2. Linear gradient for the purification of Ac-Y(OMe)DHDD-OH/-NH_2 tracked at 235 nm.

Time [min]	Flow rate [mL/min]	H_20+TFA 0.1%	ACN+TFA 0.1%
0	20	85%	15%
2	20	85%	15%
21	20	60%	40%
22	20	5%	95%
26	20	5%	95%
27.5	20	85%	15%
30	20	85%	15%

Supplementary Table 3. Linear gradient for the analysis of Ac-GHG-OH tracked at 220 nm.

Time [min]	Flow rate [mL/min]	H_20+TFA 0.1%	ACN+TFA 0.1%
0	0.4	98%	2%
15	0.4	2%	98%
16	0.4	2%	98%
17	0.4	98%	2%
20	0.4	98%	2%

Supplementary Table 4. Linear gradient for the analysis and kinetic study of Ac-Y(OMe)DHDD-OH/-NH_2.

tracked at 220 nm.

Time [min]	Flow rate [mL/min]	TEAA 25 mM	ACN:TEAA 25:75
0	0.75	80%	20%
4	0.75	80%	20%
17	0.75	40%	60%
18	0.75	5%	95%
20	0.75	5%	95%
22	0.75	80%	20%
30	0.75	80%	20%

Supplementary Table 5. Rate constants used in the kinetic model for the cycle with His, Ac-GHG-OH or Ac-Y(OMe)DHDD-NH_2 in the presence or absence of pyridine. If not stated differently the reaction rate constants were determined by the fit of the model and given with the error of the fit.

	pH 5.5 (acGHG-OH)	pH 6.5 (His)	pH 6.5 (acGHG-OH)	pH 7.5 (acGHG-OH)	pH 7.5 (acY(OMe)DHDDam)
k_0 [h^{-1}]	(6.22 ± 0.46) × 10^{-2} [a]	(4.20 ± 0.06) × 10^{-2} [a]	(4.20 ± 0.06) × 10^{-2} [a]	(3.34 ± 0.17) × 10^{-2} [a]	(3.34 ± 0.17) × 10^{-2} [a]
k_1 [L*(mmol*h)$^{-1}$]	(1.00 ± 1.65) × 10^{-5}	(1.14 ± 0.11) × 10^{-3}	(14.5 ± 1.00) × 10^{-5}	(2.47 ± 0.10) × 10^{-4}	(1.37 ± 0.21) × 10^{-3}
k_{-1} [h^{-1}]	(0.22 ± 25.2) × 10^{2}	1.79 ± 0.14	(3.89 ± 0.27) × 10^{-1}	(2.10 ± 0.10) × 10^{-1}	1.40 ± 0.18
k_2 [L*(mmol*h)$^{-1}$]	n/a	(1.58 ± 95.6) × 10^{5}	n/a	(2.02 ± 0.24) × 10^{-3}	0
k_{-2} [h^{-1}]	n/a	(2.40 ± 1.17) × 10^{-1}	n/a	(3.61 ± 0.71) × 10^{-2}	0
k_3 [L*(mmol*h)$^{-1}$]	(47.0 ± 0.96) × 10^{-6}	(8.68 ± 0.96) × 10^{-4}	(17.9 ± 0.57) × 10^{-5}	(4.89 ± 0.02) × 10^{-4}	(3.30 ± 0.37) × 10^{-4}
k_{-3} [h^{-1}]	(1.40 ± 0.16) × 10^{-2} [a]	(6.56 ± 0.43) × 10^{-2} [a]	(1.16 ± 0.13) × 10^{-2} [a]	(4.93 ± 0.58) × 10^{-3} [a]	(6.71 ± 2.01) × 10^{-3}
$t_{1/2}$ (3-pHis) [h]	40.8 ± 9.5 h	10.6 ± 0.7 h	59.7 ± 6.4 h	142.6 ± 15.4 h	n/a
k_4 [L*(mmol*h)$^{-1}$]	n/a	(2.35 ± 0.65) × 10^{-3}	n/a	(6.96 ± 60.7) × 10^{-6}	0
k_{-4} [h^{-1}]	n/a	(3.69 ± 0.2) × 10^{-1}	n/a	(0.01 ± 90.1) × 10^{-4}	0
k_{py} [L*(mmol*h)$^{-1}$]	(8.37 ± 1.12) × 10^{-3}	n/a	(1.55 ± 0.39) × 10^{-2}	(1.70 ± 0.09) × 10^{-2}	n/a
k_{-py} [h^{-1}]	(0.01 ± 1320) × 10^{8}	n/a	(0.01 ± 2450) × 10^{8}	(0.05 ± 412) × 10^{8}	n/a
k_5 [L*(mmol*h)$^{-1}$]	(0.10 ± 57.2) × 10^{-5}	n/a	811 ± 59.0	(8.52 ± 0.42) × 10^{3}	n/a

k_{-5} [L*(mmol*h)$^{-1}$]	0.09 ± 2130	n/a	(0.10 ± 76.9) × 10^{-5}	(3.54 ± 0.58) × 10^{-3}	n/a
k_{6} [L*(mmol*h)$^{-1}$]	n/a	n/a	n/a	(4.45 ± 0.30) × 10^{4}	n/a
k_{-6} [L*(mmol*h)$^{-1}$]	n/a	n/a	n/a	(1.00 ± 11.8) × 10^{-6}	n/a
k_{7} [L*(mmol*h)$^{-1}$]	(382 ± 2.75) × 10^{3}	n/a	(22.3 ± 1.12) × 10^{2}	(1.90 ± 0.89) × 10^{4}	n/a
k_{-7} [L*(mmol*h)$^{-1}$]	(2.17 ± 0.50) × 10^{-3}	n/a	(2.11 ± 0.14) × 10^{-3}	(14.0 ± 0.76) × 10^{-4}	n/a
k_{8} [L*(mmol*h)$^{-1}$]	n/a	n/a	n/a	(0.03 ± 34.7) × 10^{-4}	n/a
k_{-8} [L*(mmol*h)$^{-1}$]	n/a	n/a	n/a	(4.50 ± 0.23) × 10^{-3}	n/a

[a] empirically determined and error is the average of the absolute deviations of constants from their mean (n = 3).

Supplementary Table 6. Apparent hydrolysis of MAP in with 75 mM different amino acids in a 500 mM MOPS buffered solution. The error is the average of the absolute deviations of constants from their mean (n = 3).

Amino acid	Apparent hydrolysis constant of MAP k'_0 [h^{-1}]
His	(2.34 ± 0.11) × 10^{-1}
Arg	(4.71 ± 0.05) × 10^{-2}
Lys	(4.70 ± 0.02) × 10^{-2}
Cys	(4.80 ± 0.07) × 10^{-2}
Ser	(4.80 ± 0.07) × 10^{-2}
Tyr[a]	(9.00 ± 4.67) × 10^{-4}
Asp	(4.88 ± 0.14) × 10^{-2}

[a]sodium carbonate buffer at pH 10.5.

Supplementary Table 7. Apparent first-order constants for hydrolysis $k'_{3\text{-pHis}}$ ([h^{-1}]) of 3-pHis in the presence of pyridine. The error is the average of the absolute deviations of constants from their mean (n = 3).

Concentration of pyridine [mM]	5.5	6.5	7.5
5	(4.50 ± 0.80) × 10^{-2}	(1.83 ± 0.10) × 10^{-2}	(9.03 ± 1.62) × 10^{-3}
$t_{1/2}$ (3-pHis) [h]	15.9 ± 2.4 h	37.9 ± 2.0 h	80.2 ± 16.6 h
15	n/a	(3.55 ± 0.86) × 10^{-2}	(2.45 ± 0.02) × 10^{-2}
$t_{1/2}$ (3-pHis) [h]		20.7 ± 5.0 h	28.3 ± 0.2 h
25	n/a	(7.27 ± 0.31) × 10^{-2}	(4.36 ± 0.69) × 10^{-2}
$t_{1/2}$ (3-pHis) [h]		9.56 ± 0.4 h	16.3 ± 2.3 h

Supplementary Table 8. Sequences of the non-functional structures.

ssDNA	$(AGTC)_8$
dsDNA	$(ATCG)_8$
RNA	U_{30}
pSS	109 monomer units, 22.5 kDa

III. Supplementary Figures

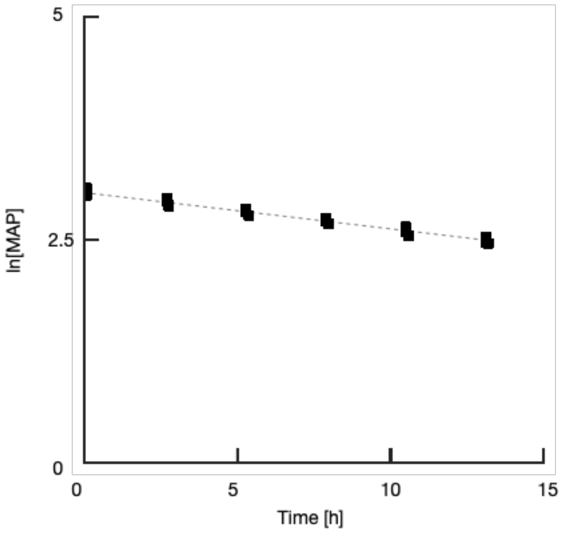

Supplementary Fig. 1. Representative empirical determination of the pseudo-first-order hydrolysis of 20 mM MAP in a 500 mM MES buffered solution at pH 6.5. (n = 3)

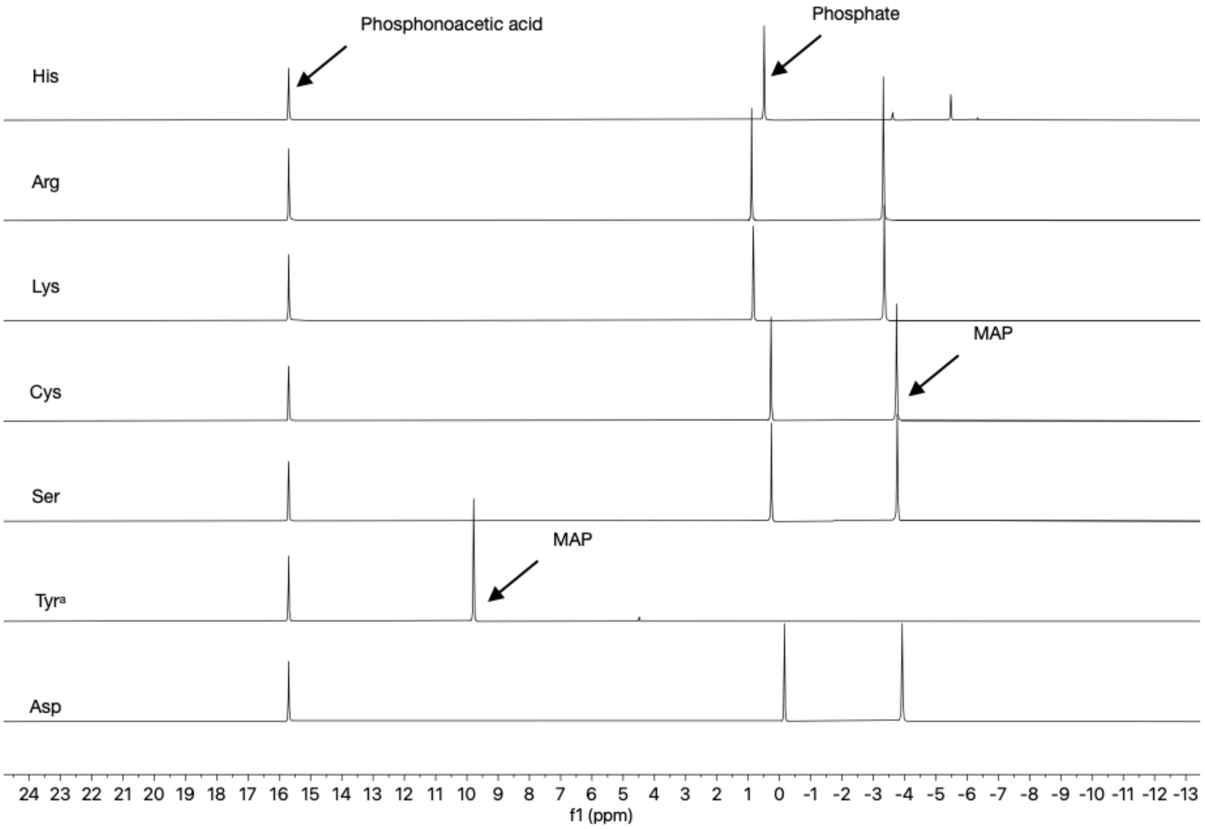

Supplementary Fig. 2. Representative ^{31}P-NMR spectra of the consumption of MAP in the presence of different amino acids after 60 h (75 mM amino acid and 80 mM MAP in 500 mM MES buffered solution pH 6.5. 60 mM Tyr and 80 mM MAP in 500 mM sodium carbonate solution pH 10.5).

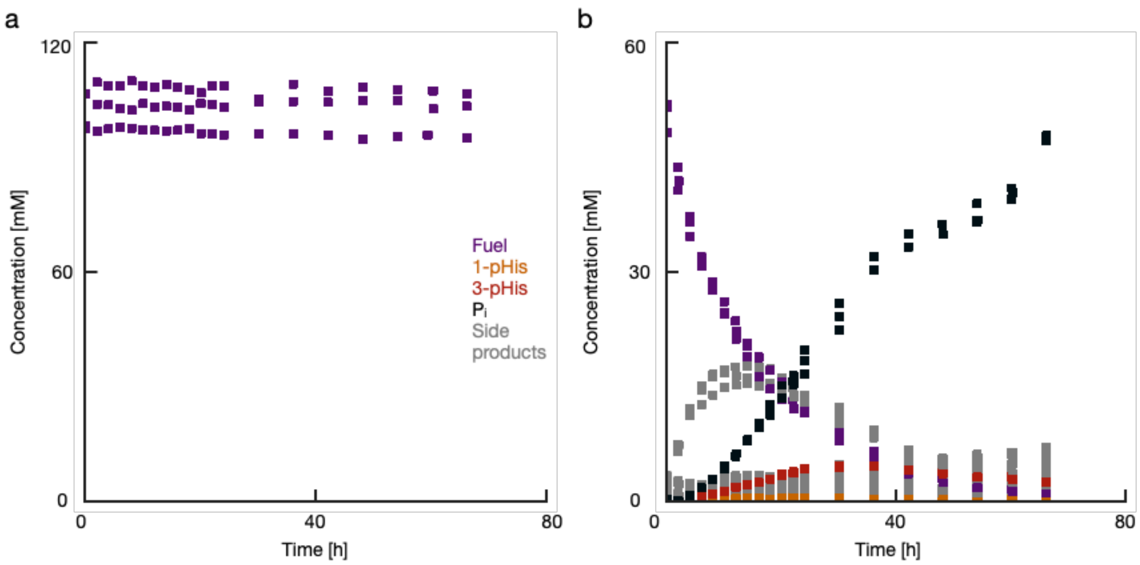

Supplementary Fig. 3. Investigation of different biologically relevant fuels (purple) **a** trimetaphosphate (TMP) and **b** diamidophosphate (DAP). Concentration profiles of the phosphorous-containing compounds were recorded with ^{31}P-NMR. **a** Phosphorylation of His in the presence of TMP (purple). No conversion (75 mM His, 100 mM TMP in a 500 mM MES buffered solution pH 6.5). **b** Phosphorylation of His in the presence of DAP (purple). 1-pHis is highlighted in yellow, 3-pHis in red (75 mM His , 50 mM MAP in a 500 mM MES buffered solution pH 6.5).

The hydrolysis constant of DAP in a 500 mM MES buffered solution at pH = 6.5 is k_0 = (3.97 ± 0.91) × 10^{-3} h^{-1}, whereas the apparent hydrolysis constant in the presence of 75 mM His k'_0 = (5.65 ± 0.13) × 10^{-2} h^{-1} is increased.

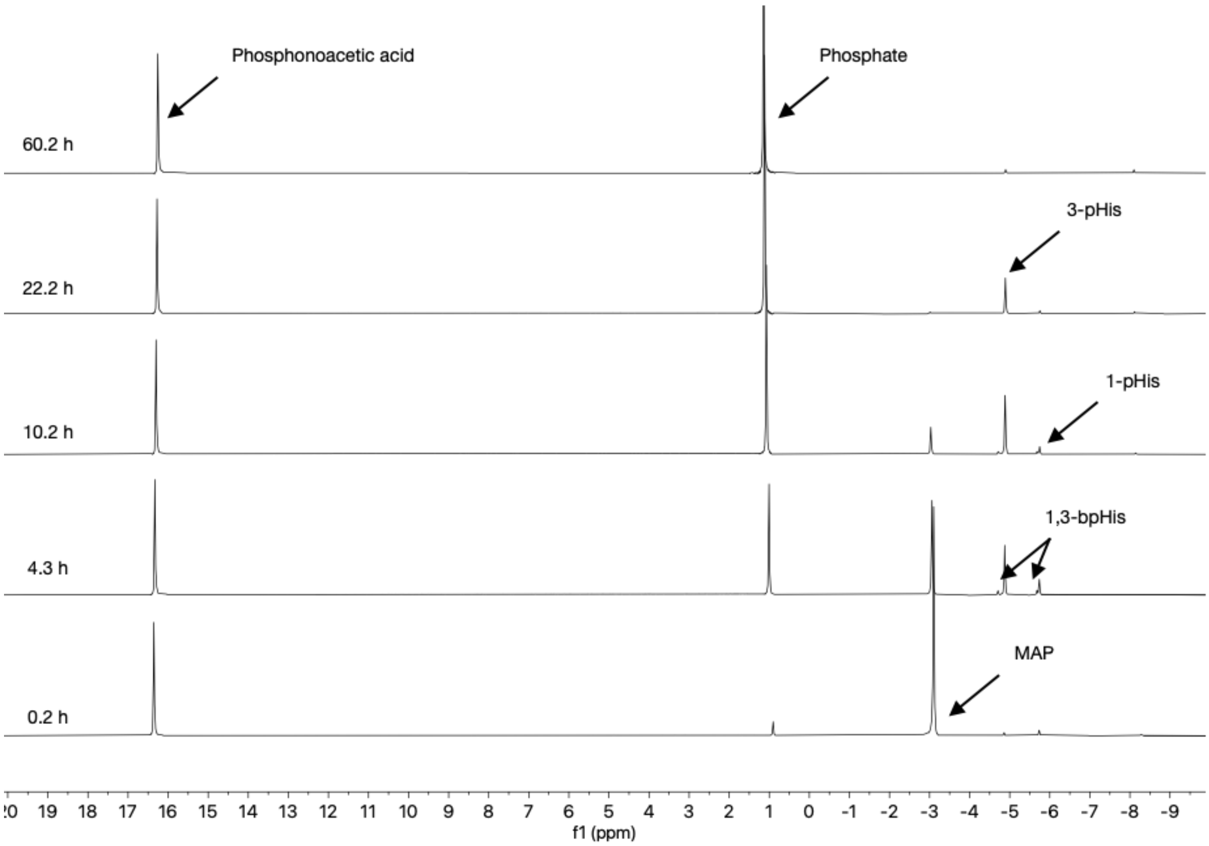

Supplementary Fig. 4. Representative time evolution of the chemical shifts in the ^{31}P-NMR spectra of the phosphorylation of His at the expense of MAP (75 mM His and 80 mM MAP in a 500 mM MES buffered solution pH 6.5).

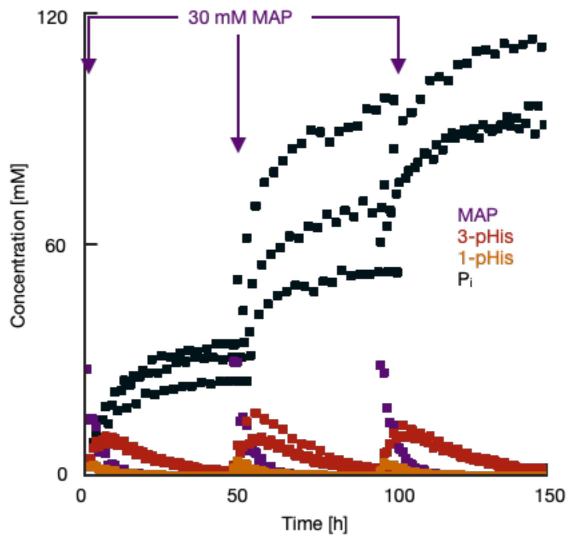

Supplementary Fig. 5. Concentration profiles of the recycling experiment of 75 mM His fueled with 30 mM MAP each time at pH 6.5 in a 500 mM MES buffered solution at pH 6.5 recorded with ^{31}P-NMR.

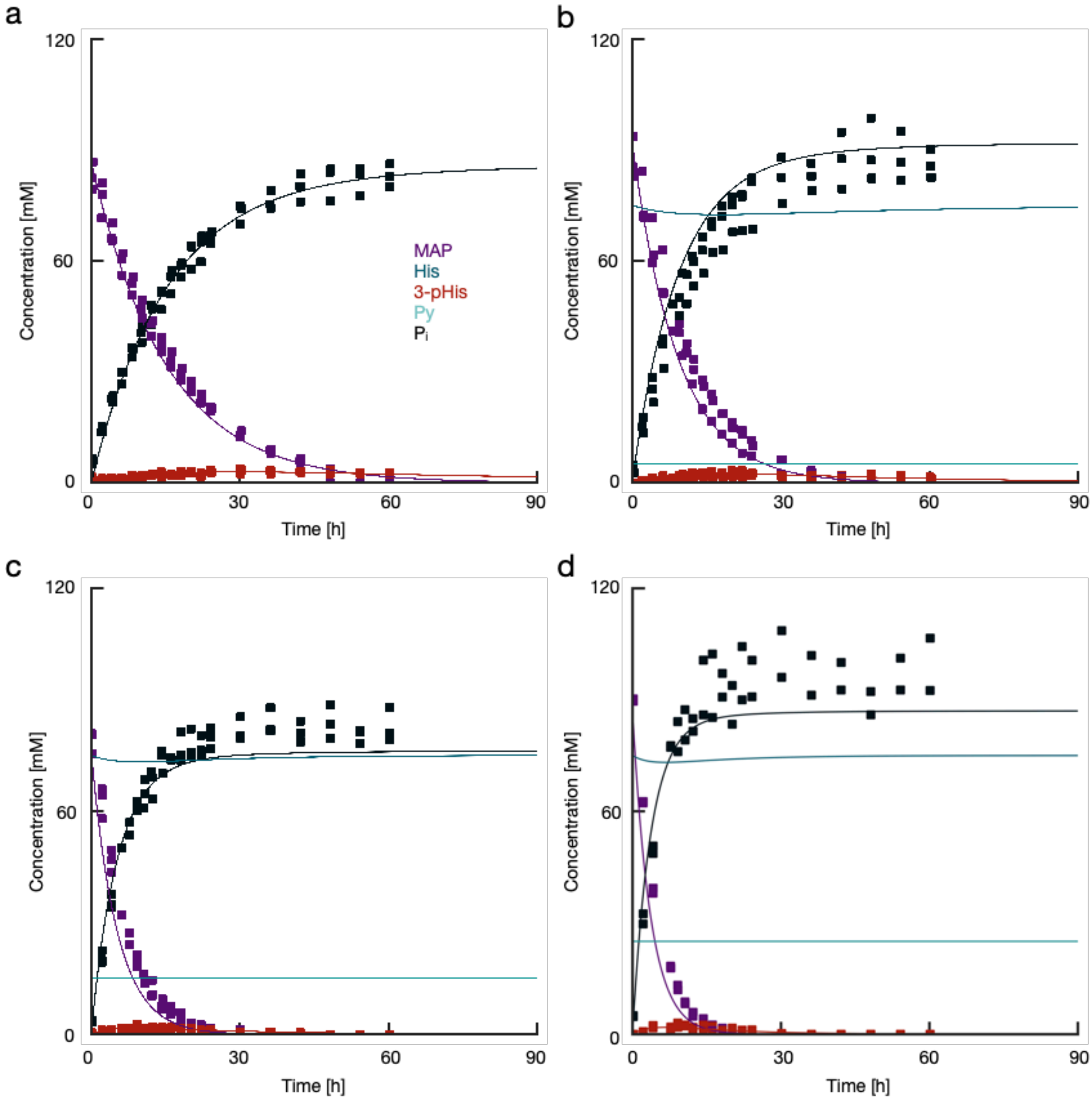

Supplementary Fig. 6. Concentration profiles (squares) and kinetic profiles (lines) of the phosphorylation cycle of Ac-GHG-OH with MAP at pH 5.5 and following pyridine concentrations **a** 0, **b** 5, **c** 15, and **d** 25 mM recorded with ^{31}P-NMR (75 mM His, 80 mM MAP, x mM pyridine in a 500 mM MES buffered solution at pH 5.5).

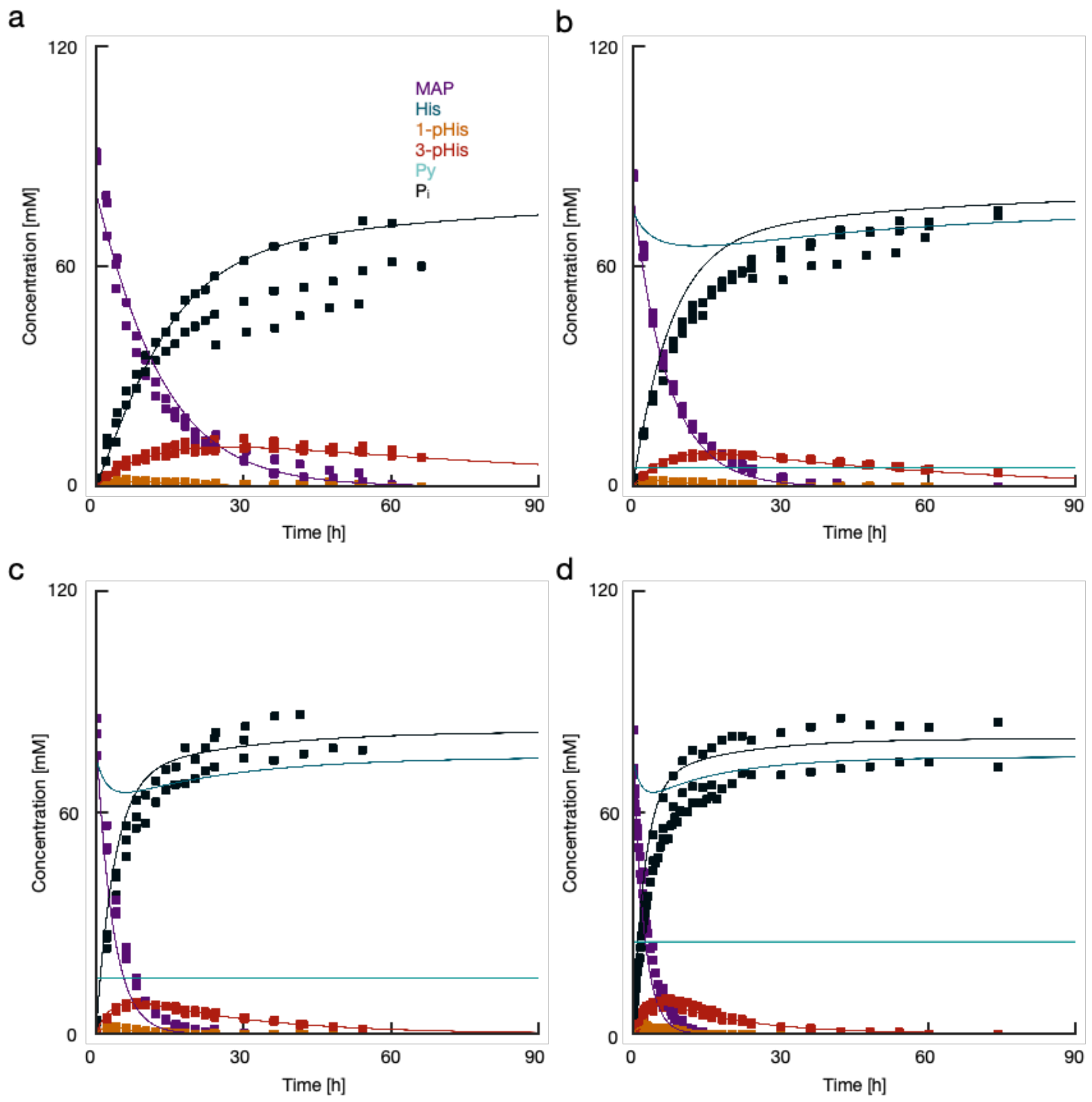

Supplementary Fig. 7. Concentration profiles (squares) and kinetic profiles (lines) of the phosphorylation cycle of Ac-GHG-OH with MAP at pH 6.5 and following pyridine concentrations **a** 0, **b** 5, **c**15, and **d** 25 mM recorded with ^{31}P-NMR (75 mM His, 80 mM MAP, x mM pyridine in a 500 mM MES buffered solution at pH 6.5).

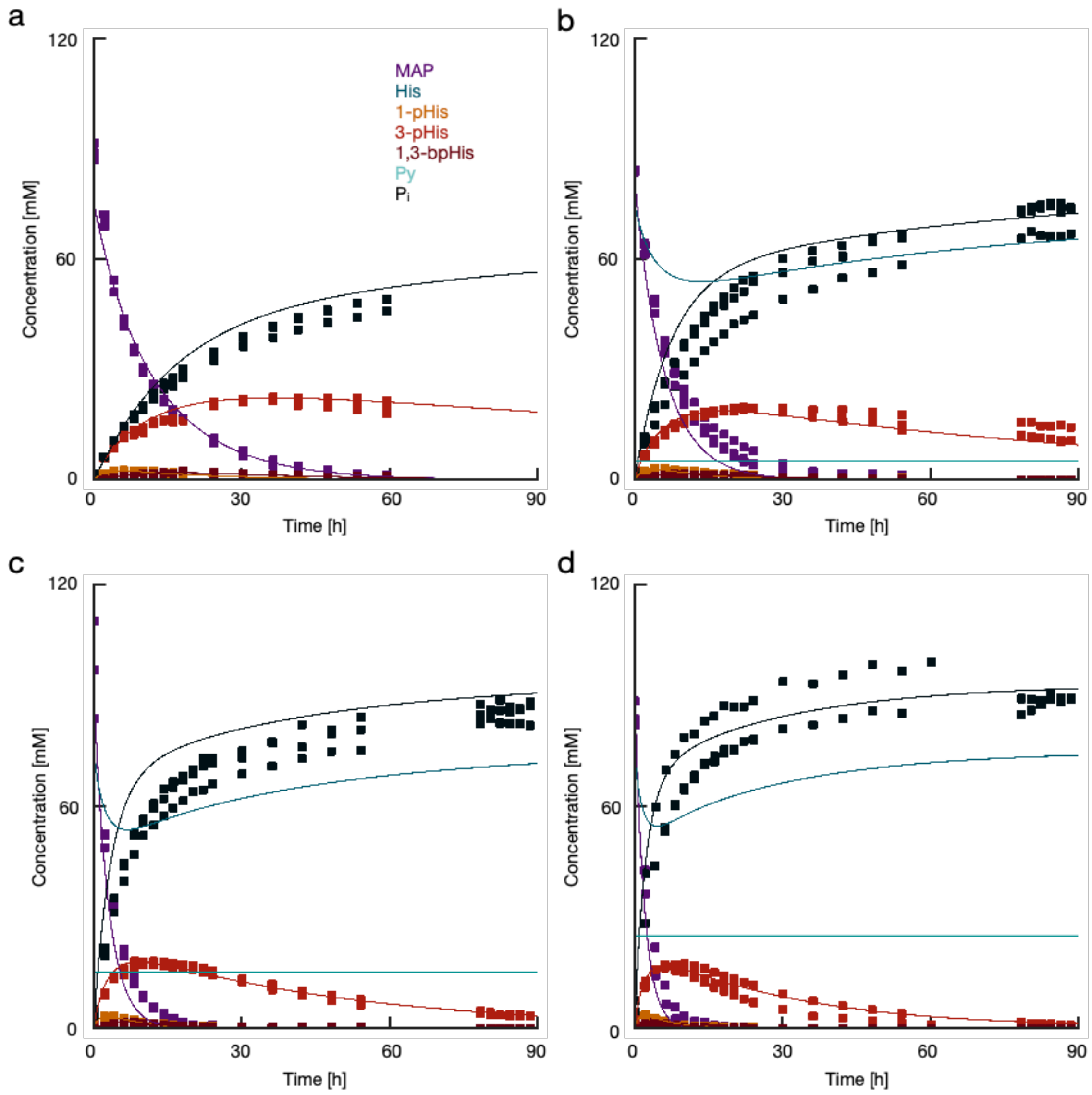

Supplementary Fig. 8. Concentration profiles (squares) and kinetic profiles (lines) of the phosphorylation cycle of Ac-GHG-OH with MAP at pH 7.5 and following pyridine concentrations **a** 0, **b** 5, **c**15, and **d** 25 mM recorded with ^{31}P-NMR (75 mM His, 80 mM MAP, x mM pyridine in a 500 mM MOPS buffered solution at pH 7.5).

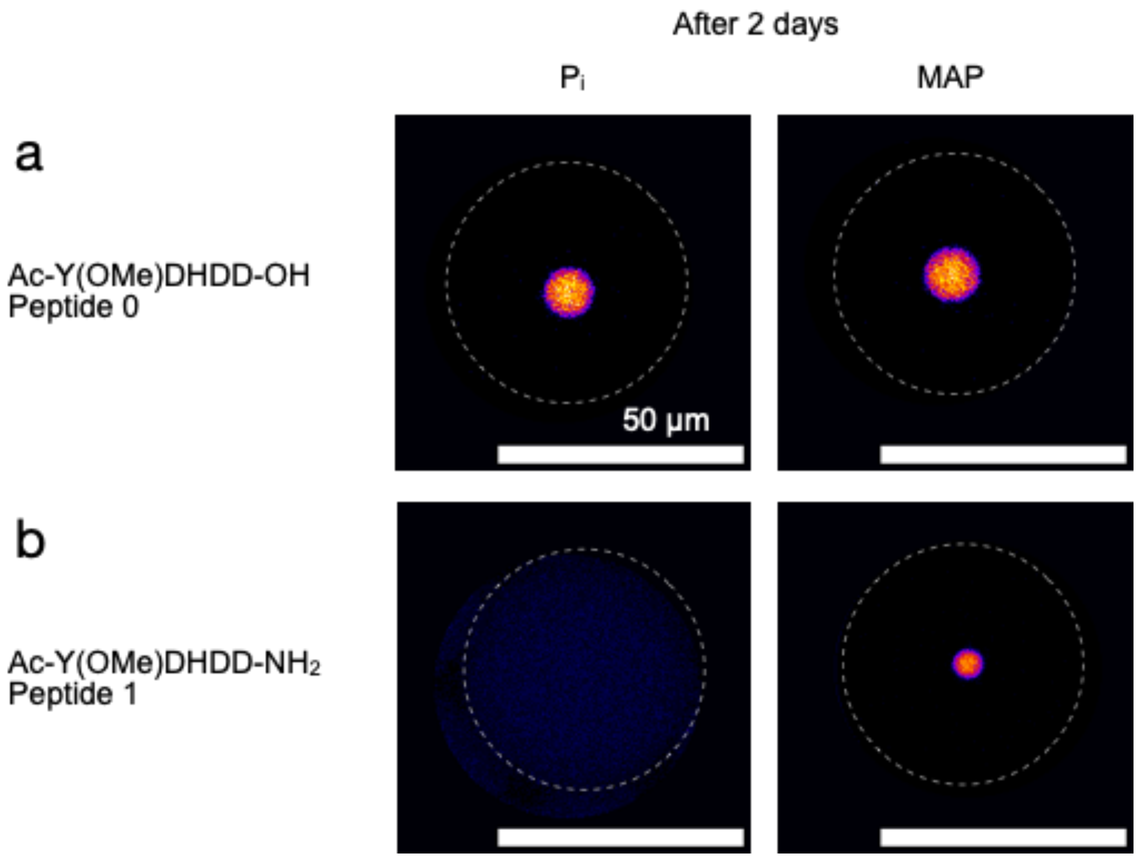

Supplementary Fig. 9. Design of the peptide for complex coacervation formation. The dotted line represents the microreactor, and the micrographs were recorded after 2 days. **a** Ac-Y(OMe)DHDD-OH forms droplets with phosphate or MAP. **b** Ac-Y(OMe)DHDD-NH_2 forms droplets only with MAP. (15 mM Ac-Y(OMe)DHDD-OH/20 mM Ac-Y(OMe)DHDD-NH_2, 12.5 mM P_i/MAP and 50 mM R_{30} (charges) in a 100 mM MOPS buffered solution at pH 7.5).

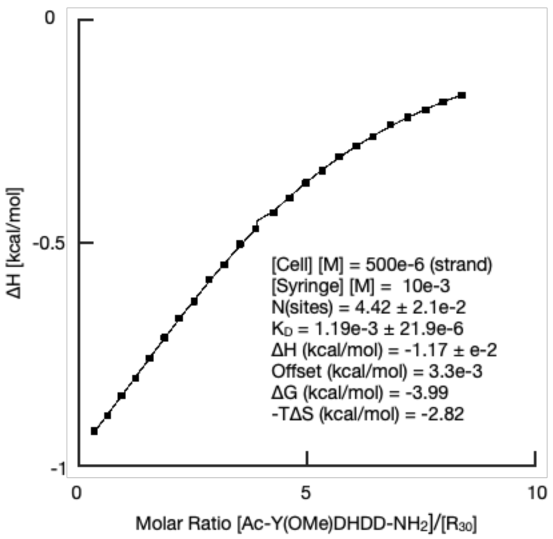

Supplementary Fig. 10. ITC titration curve given in enthalpy change versus the molar ratio.

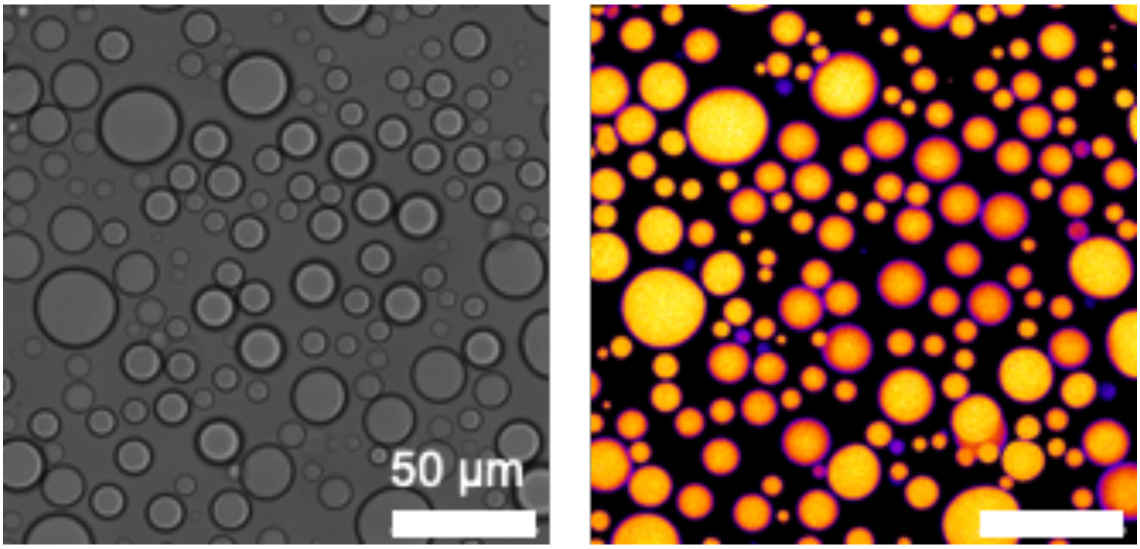

Supplementary Fig. 11. Representative micrographs of complex coacervate droplets outside microreactors 6.5 h after fueling. The left micrograph shows the maximum z-projection of a z-stack with a pseudocolor-coding of the emission channel, and the right one shows the brightfield (20 mM Ac-Y(OMe)DHDD-NH_2, 50 mM MAP and 50 mM R_{30} (charges) in a 75 mM MOPS buffered solution at pH 7.5).

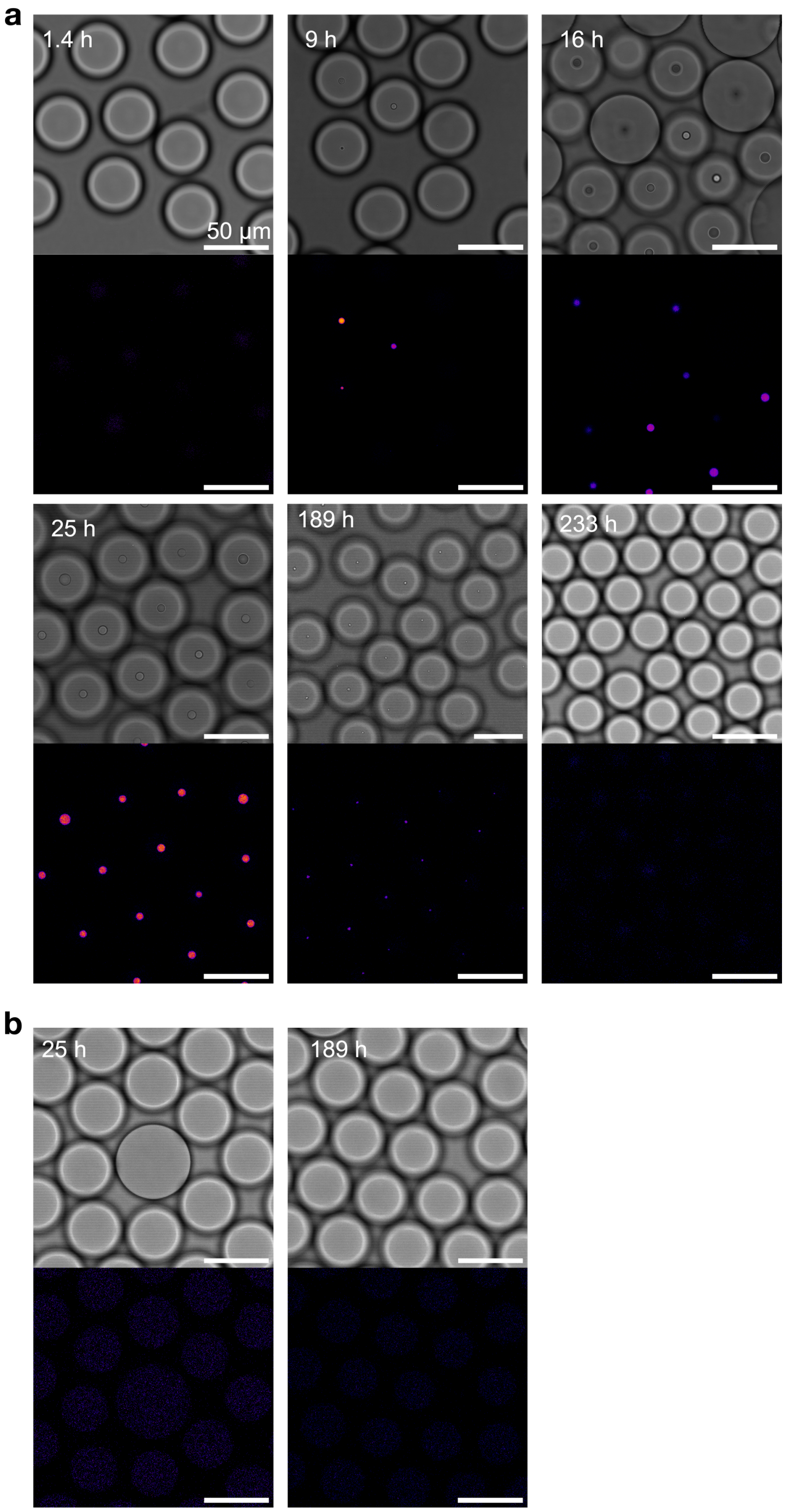

Supplementary Fig. 12. Representative micrographs of multiple microreactors over time. The top micrographs show the brightfield, and the bottom one shows the maximum z-projection of a z-stack with a pseudocolor-coding of the emission channel (20 mM pep1, 12.5 mM MAP, 50 mM R_{30} (charges), 200 nM sulforhodamine B, 500 nM Cy5-R_{30}, 100 mM MOPS pH 7.5). **a** Emerging, growing, and dissolution of complex coacervate droplets over time. **b** Control microreactors with 12.5 mM P_i instead of MAP over time.

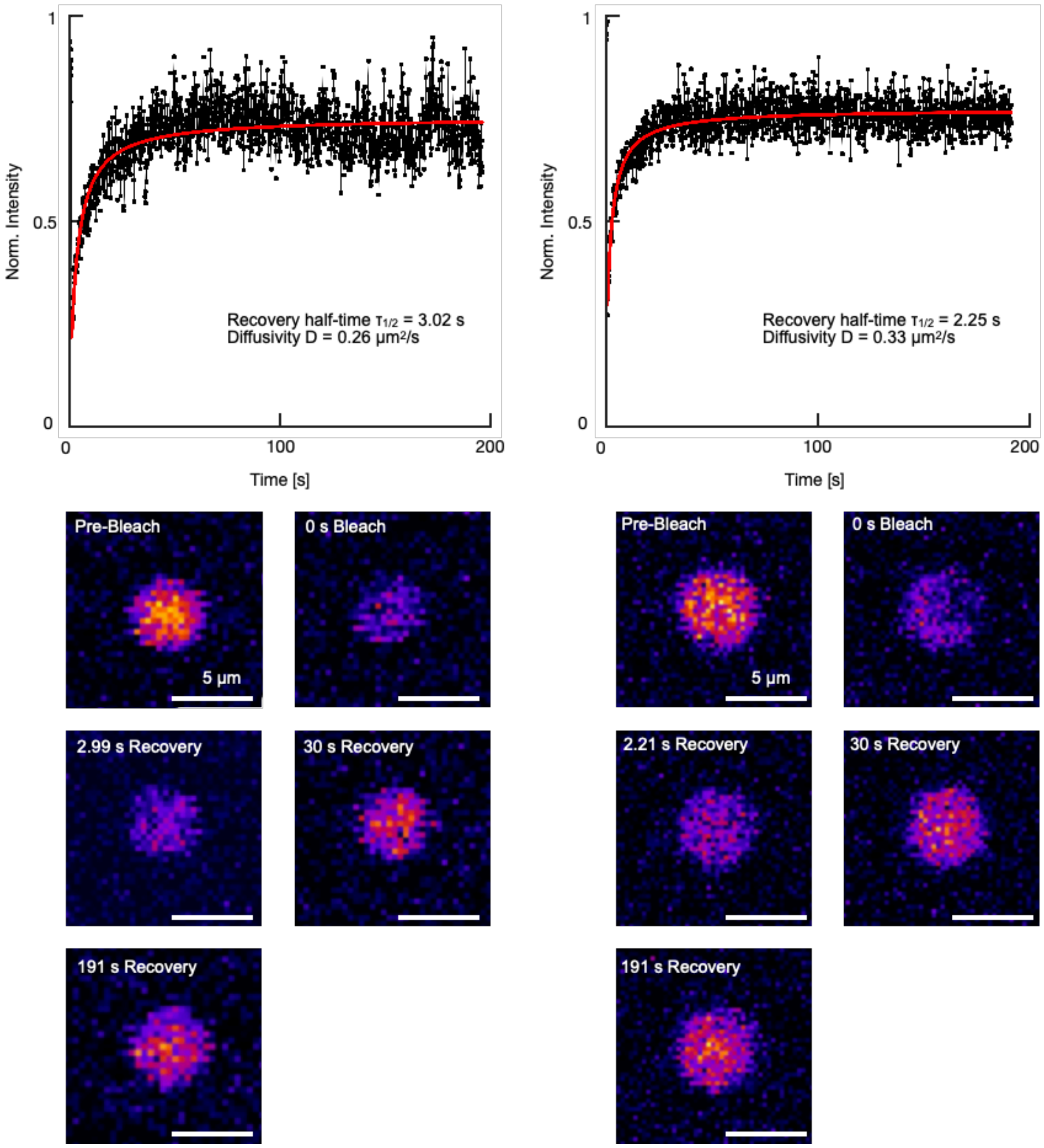

Supplementary Fig. 13. Representative FRAP data 3 days after the start of the reaction cycle *(*20 mM pep1, 12.5 mM MAP, 50 mM R_{30} (charges), 200 nM sulforhodamine B, 500 nM Cy5-R_{30}, 100 mM MOPS pH 7.5*)*.

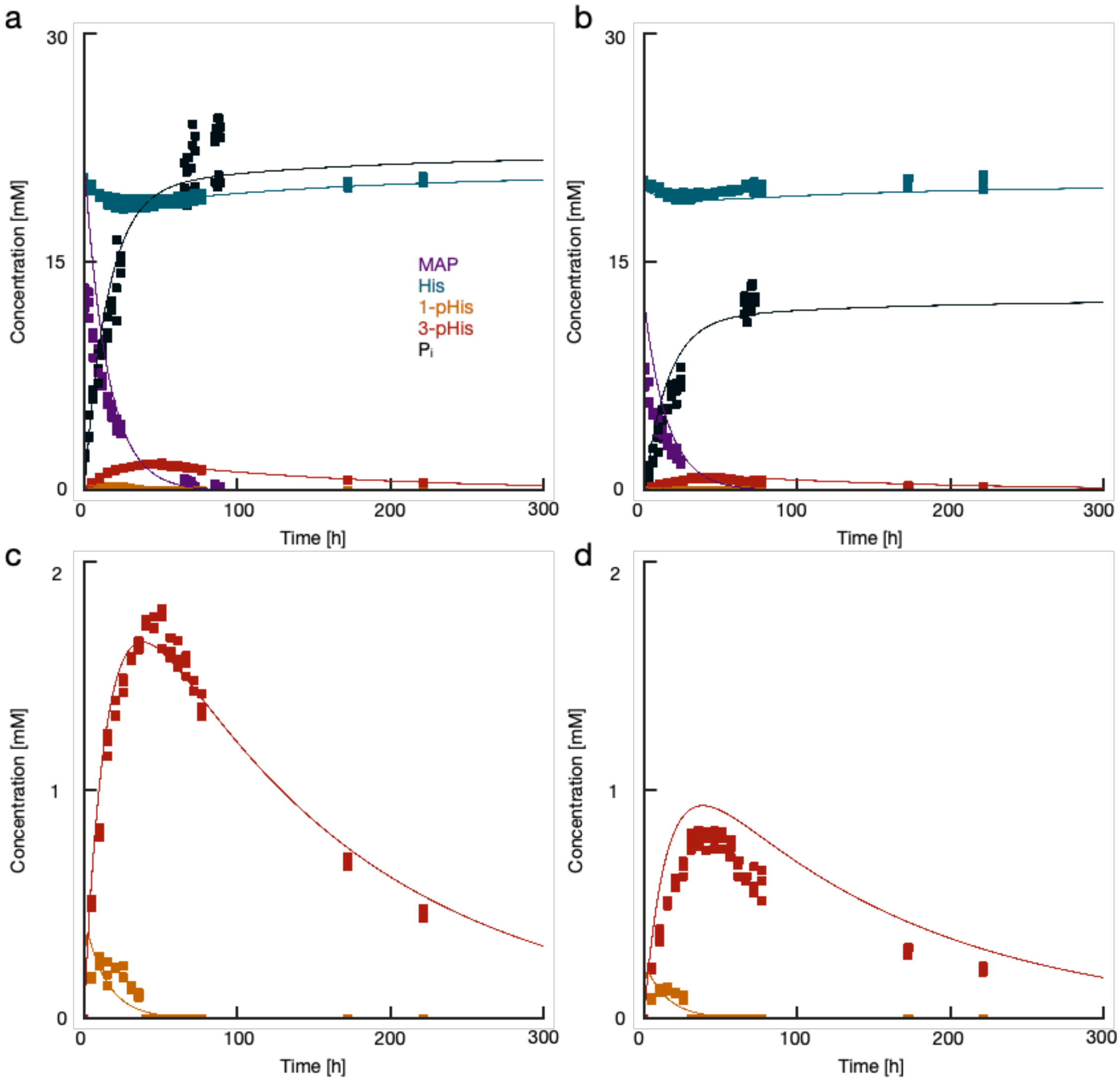

Supplementary Fig. 14. Concentration profiles (squares) and kinetic profiles (lines) of the phosphorylation cycle of 20 mM Ac-Y(OMe)DHDD-NH_2 with **a** and **c** 25 mM and **b** and **d** 12.5 mM MAP in a 100 mM MOPS buffered solution at pH 7.5 followed with ^{31}P-NMR (MAP and P_i) and analytical HPLC (His, 1-pHis, and 3-pHis). **c** and **d** Magnification of the profiles 1- and 3-pHis.

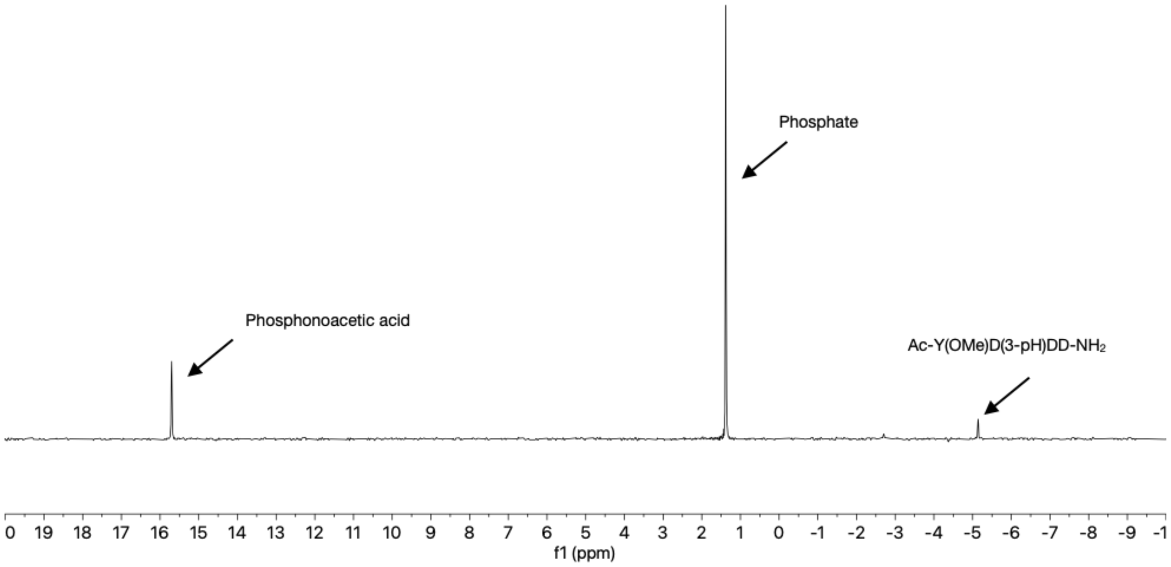

Supplementary Fig. 15. Representative ^{31}P-NMR spectra of Ac-Y(OMe)D(3-pHis)DD-NH_2 60 h after the start of the reaction (spectrum was recorded with 15 s relaxation delay and 256 scans; 20 mM pep1, 12.5 mM MAP, 100 mM MOPS pH 7.5*)*.

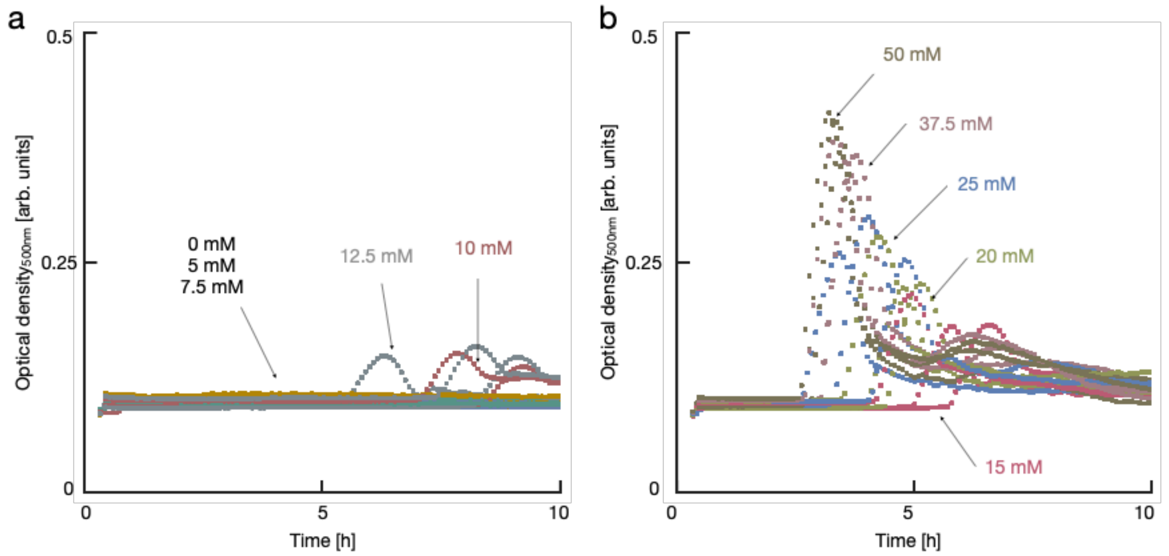

Supplementary Fig. 16. Absorbance profiles to determine the lag time and the maximum intensity dependent on fuel concentration. 20 mM Ac-Y(OMe)DHDD-NH_2, 50 mM R_{30}, 2.5% PEG_{8000}, **a** 0, 5, 7.5, 10, 12.5 mM **b** 15, 20, 25, 37.5, 50 mM MAP, (12.5+x) mM P_i, (100-x) mM MOPS pH 7.5. (n = 3).

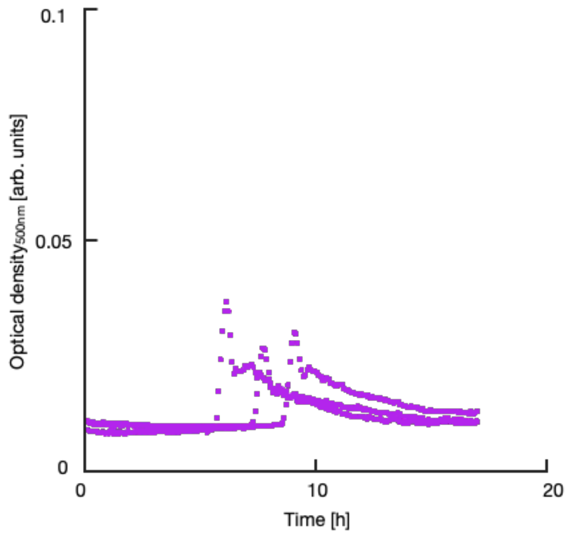

Supplementary Fig. 17. Absorbance profile of the refueling experiment of 20 mM Ac-Y(OMe)DHDD-NH_2, 50 mM R_{30}, 2.5% PEG_{8000}, fueled with 12.5 mM MAP at pH 7.5 in a 100 mM MOPS buffered solution after 7 days.

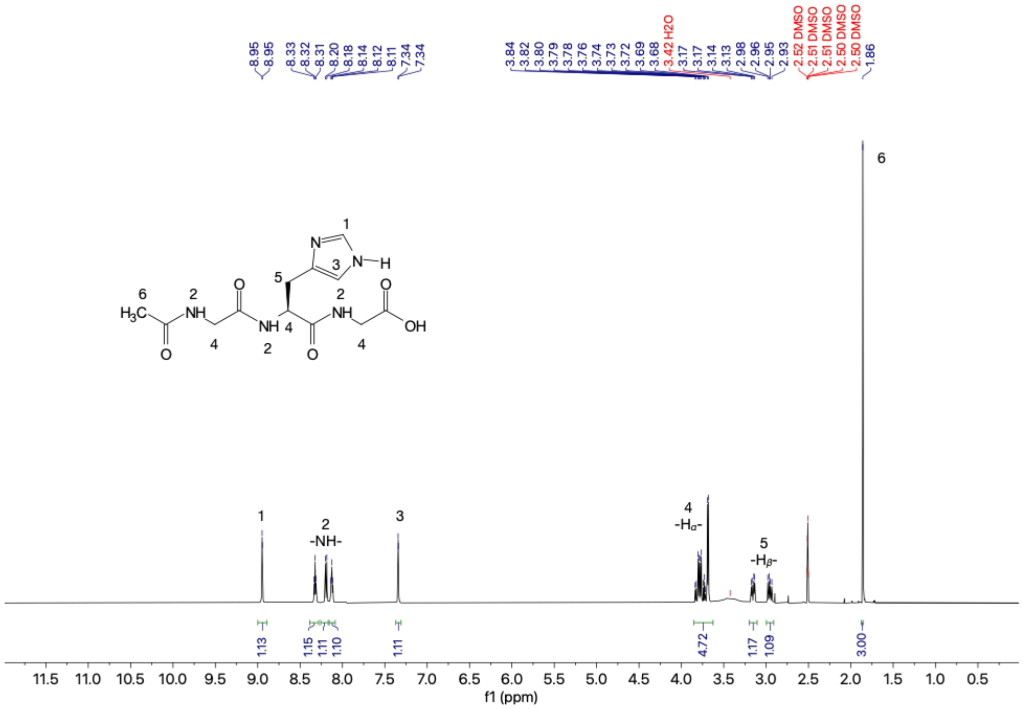

Supplementary Fig. 18. ^{1}H-NMR spectrum of Ac-GHG-OH. Spectrum was recorded on a Bruker AV-HD500 NMR-spectrometer at 500 MHz with 5 s relaxation delay and 32 scans.

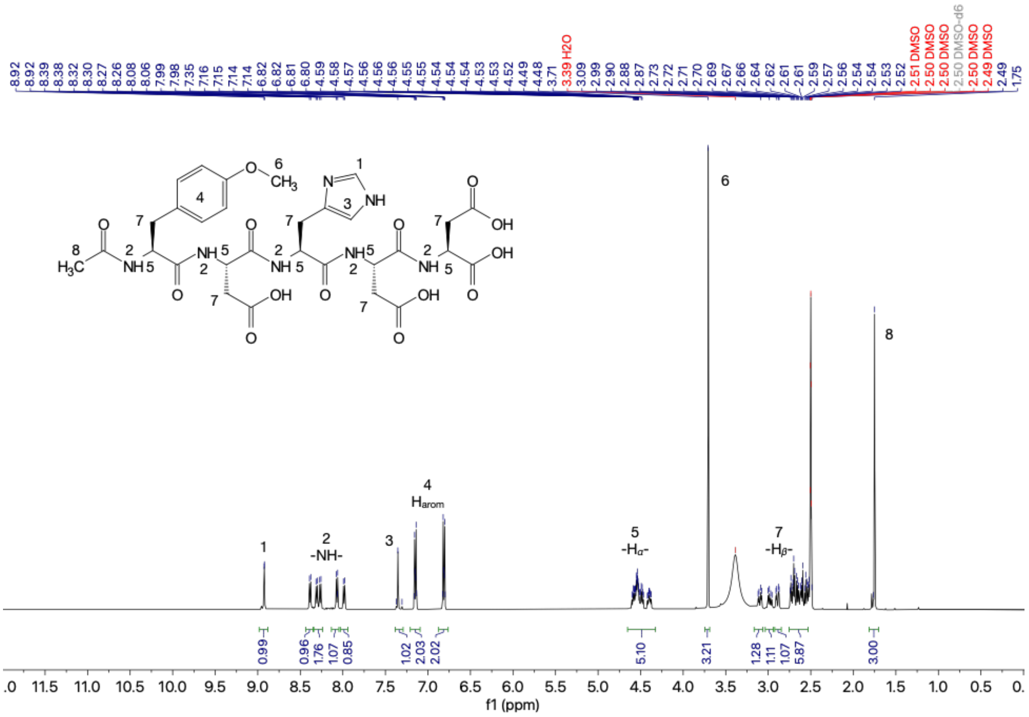

Supplementary Fig. 19. ^{1}H-NMR spectrum of Ac-Y(OMe)DHDD-OH. Spectrum was recorded on a Bruker AV-HD500 NMR-spectrometer at 500 MHz with 5 s relaxation delay and 32 scans.

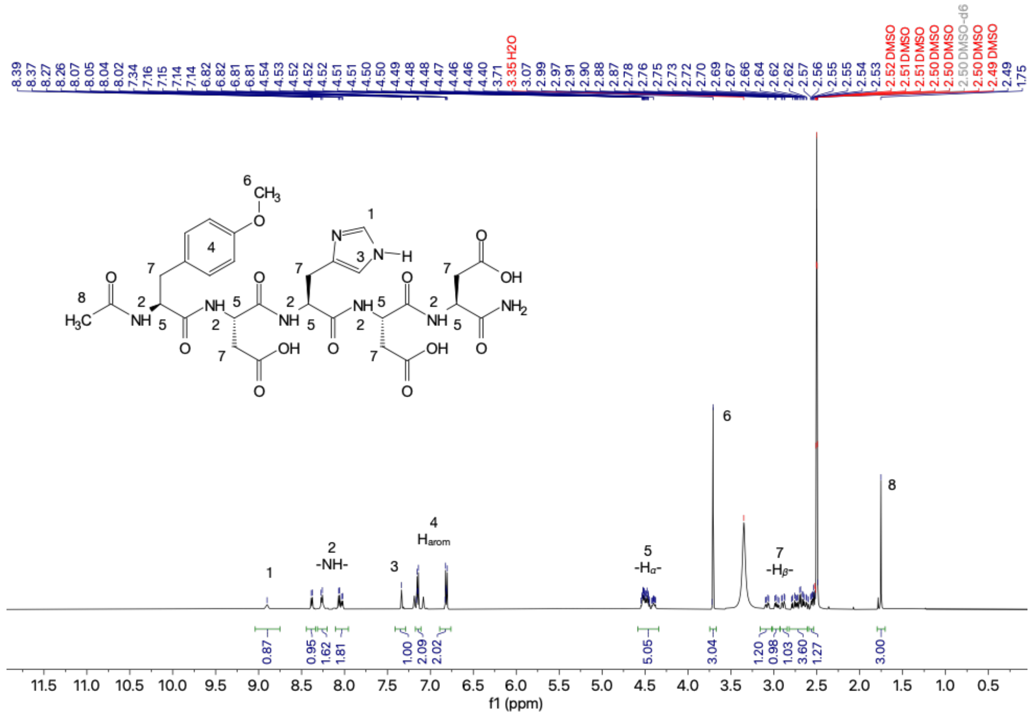

Supplementary Fig. 20. ^{1}H-NMR spectrum of Ac-Y(OMe)DHDD-NH_2. Spectrum was recorded on a Bruker AV-HD500 NMR-spectrometer at 500 MHz with 5 s relaxation delay and 32 scans.
