# Supplementary material for: Active droplets through enzyme-free, dynamic phosphorylation
Source: Nat Commun. 2024 May 17;15:4204. doi: 10.1038/s41467-024-48571-z (PMC11101487; doi:10.1038/s41467-024-48571-z)
Supplement: Supplementary file 3 — Description of Additional Supplementary Files [file 41467_2024_48571_MOESM3_ESM.pdf]

Description of Additional Supplementary Files

Supplementary Movie 1: Fusion of droplets after 8.5 h in a representative microreactor. The micrographs are a maximum z-projection of a z-stack with a pseudocolor-coding and the scale bar is 10 μm (20 mM peptide 1, 12.5 mM MAP, 50 mM R_{30} (charges) and 200 nM sulforhodamine B in 100 mM MOPS buffer at pH = 7.5).
